# Galvano Therapeutics

**Published:** 1874-07

**Authors:** 


The report on Galvano-Therapeutics to the Illinois State Medical Society is from the pen of Dr. David Prince, although the title page omits to mention this fact. It is an interesting collection of clinical observations in the Uses of Electricity and of the recent advances in its application. The report does not, of course, pretend to embrace the entire field, but simply gives a concise relation of certain facts in several of the departments of Galvano-Therapeutics. Dr. Prince is well known as a medical writer by those who have had the pleasure of reading his work, entitled "Plastics and Orthopedics" in the present essay he has fully sustained his reputation as a thorough careful observer.

